# Retention in a low‐resource, high‐burden South African cohort on antiretroviral therapy: Retrospective, longitudinal analysis comparing six measures of retention

**DOI:** 10.1002/jia2.70046

**Published:** 2025-10-14

**Authors:** Claire M. Keene, Jonathan Euvrard, Tamsin K. Phillips, Mike English, Jacob McKnight, Catherine Orrell

**Affiliations:** ^1^ Centre for Global Health Research Nuffield Department of Medicine University of Oxford Oxford UK; ^2^ Centre for Integrated Data and Epidemiological Research School of Public Health University of Cape Town Cape Town South Africa; ^3^ Division of Epidemiology and Biostatistics School of Public Health University of Cape Town Cape Town South Africa; ^4^ Department of Medicine Faculty of Health Sciences University of Cape Town Cape Town South Africa; ^5^ Institute of Infectious Disease Desmond Tutu HIV Centre Cape Town South Africa

**Keywords:** antiretroviral therapy, engagement, longitudinal, measurement, retention

## Abstract

**Introduction:**

Retention on antiretroviral therapy (ART) is a prerequisite for adherence and subsequent treatment success. Measuring retention is also easily implementable at facility and population levels, making it pragmatic to monitor ART programme success. However, despite its ubiquitous global use, there is little consistency in the measurement of retention.

**Methods:**

This study retrospectively applied six measures of retention to one cohort of adults (initiating ART after 01‐09‐2016, with ≥1 year of observation time to database closure on 30‐09‐2022), in a low‐resource, high HIV‐burden setting in South Africa. Using routine healthcare data from the Western Cape's Provincial Health Data Centre, loss to follow‐up (LTFU), fixed‐point retention, visit constancy, visit gaps, treatment interruptions and medication possession ratio (MPR) were described over 5 years from initiation. Individuals were considered “continuously retained” if they did not experience attrition throughout their observed follow‐up. Measures were compared using the proportion misassigned and Cohen's Kappa statistic.

**Results:**

The median age of the cohort (*n* = 68,888) was 31 years (interquartile range [IQR] 26–38) at initiation, with 69% (47,631/68,888) female, and a median observed follow‐up of 4 years (IQR 3–5). Across different measures, retention was low, and declined over time. There was variable overlap; the proportion continuously retained throughout their observed follow‐up ranged from 60% (41,268/68,888 not LTFU) to 32% (22,381/68,888 MPR ≥80%). Retention by all measures was strongly associated with viral suppression.

**Conclusions:**

By all measures, large proportions of people in this setting were considered out of ART care during 5 years of observed follow‐up time from initiation. This makes retention a critical target for intervention to improve population‐level viral suppression and achieve epidemic control. Measuring longitudinal retention revealed that most people disengaged from ART care at some point after initiation. Certain measures of retention (e.g. treatment interruptions) identified people in and out of care with more granularity, whereas blunter measures (e.g. LTFU) misassigned individuals’ retention status and missed patterns of retention over time as people cycled in and out of care between points of measurement. Ultimately, the choice of measure depends on the purpose of the evaluation and on the data available, but, where possible, more granular measures are recommended.

## 1 INTRODUCTION

Retention in HIV care is a prerequisite for successful treatment outcomes: individuals must be retained in order to receive antiretroviral therapy (ART) refills, so they can be adherent and ultimately suppress their viral load (VL) [1, 2, 3, 4]. Retention is reflective of an individual's engagement with the health services and the facility, so improvements reflect the success of interventions that address service delivery and organization of care [5, 6]. Additionally, measuring the retention dimension is easily implementable, making it a pragmatic option to monitor ART programmes [4, 7].

Despite recognition that retention is an important dimension of engagement, measures of retention vary widely across different agencies, programmes and research studies [4]. A recent scoping study identified 18 measures to evaluate retention in a sub‐Saharan African context, but found little standardization of measures used between studies, and wide variation in the thresholds that considered people “in care,” which can lead to different conclusions [8].

Therefore, this study applied a range of metrics to measure retention over time from ART initiation in a cohort of people living with HIV (PWH) who initiated treatment in the universal test‐and‐treat era in a low‐resource, high‐burden setting. Longitudinal analyses can complement cross‐sectional insights with more nuanced descriptions of the continuity of retention over time [2, 9, 10, 11, 12].

The aim was twofold: to describe retention over 5 years from ART initiation and the association of continuous retention (no attrition throughout their observed follow‐up) with viral suppression; and to directly compare different measures of retention and the impact on inferences drawn from the results.

## 2 METHODS

### 2.1 Study design

This study was a retrospective, observational cohort analysis applying six measures of retention to one cohort of adults on ART in Khayelitsha and Gugulethu, South Africa. The protocol is available on the Oxford University Research Archive [13]. Ethical approval for this study was obtained from the University of Cape Town's Human Research Ethics Committee (066/2022). Findings were reported according to the Strengthening the Reporting of Observational Studies in Epidemiology Cohort Studies checklist [14].

### 2.2 Setting, data and cohort eligibility

Khayelitsha and Gugulethu are two low‐resource areas in the Western Cape Province of South Africa with large, well‐established cohorts on ART [15]. Both have high HIV prevalence alongside high rates of non‐communicable disease, poverty, unemployment, violence and mobility for work, making retention in ART care particularly challenging [16, 17]. Routine ART dispensing is generally every 2 months, through primary care clinics or differentiated models like facility‐ or community‐based adherence clubs. Programmes to support retention include efforts to trace people out of care through phone calls and the national “Welcome Back” campaign. Many of these programmes receive international funding and are delivered through implementing non‐governmental organizations (NGOs). A “Welcome Service” to support sustained re‐engagement was also developed and implemented with support from Médecins Sans Frontières in Khayelitsha during this period [18].

Demographic, mortality, dispensing and laboratory data from across all facilities in the Western Cape were obtained from the Provincial Health Data Centre (PHDC, Supporting Information 1a). The PHDC is a data repository of linked, individual‐level health information from existing governmental routine health data systems, consolidated and curated by the Western Cape Department of Health and Wellness [19]. Data includes all visits to facilities within the province, so retention can be evaluated regardless of which facility an individual attends.

Adults (≥15 years and ≤85 years old) who initiated ART between 01‐09‐2016 (implementation of universal test‐and‐treat guidance) and 30‐09‐2021 (with ≥1 year possible observation time before the database closure on 30‐09‐2022) and ever sought care in Khayelitsha or Gugulethu (regardless of where they initiated) were included in the analysis (Supporting Information 1b). Censoring occurred at death or database closure.

### 2.3 Variables and analysis

#### 2.3.1 Variables

*Longitudinal retention*: Retention was measured using pharmacy‐refill data to indicate interactions with ART services. A sensitivity analysis combined pharmacy‐refill visits with data on other visits, including ART clinic visits and HIV‐related laboratory test results, to indicate interactions with broader HIV services beyond ART collection. Measures of retention were selected according to the following criteria:

- Identified in our 2022 scoping study of measures of engagement with HIV care in sub‐Saharan Africa: 18 candidate measures of retention identified as “engagement with ART services” [8].
- Calculable from PHDC data (Supporting Information 1a) [19], including pharmacy refills, visits to facilities and laboratory results.
- Demonstrated association with treatment outcomes (such as VL) in the literature [20].
- Commonly used in practice (based on study team and stakeholder input).

**Figure 1:**
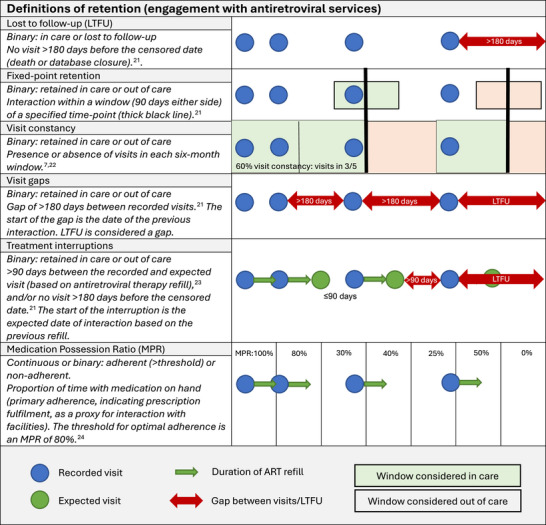
Definitions of measures of retention, illustrated using the same four hypothetical visits over a period of observed follow‐up time.

The six measures used in this study are defined and described in Figure 1. Individuals were considered “continuously retained” if they did not disengage throughout their observed follow‐up.

The optimal threshold to consider an individual meaningfully out of care is not clear in the literature; longer thresholds are more strongly associated with VL outcomes [21], but risk missing those out of care who warrant intervention. A threshold of 180 days without a visit was used for loss to follow‐up (LTFU), fixed‐point retention (90 days either side of the fixed‐point), visit constancy and visit gaps, as this has been widely used [22, 23, 24, 25, 26], is strongly associated with viraemia [7], and results in less misclassification [27]. A substantial treatment interruption was defined as >90 days late for an expected visit, as the 2023 South African National Department of Health Antiretroviral Therapy Guidelines uses this threshold to direct management of returning PWH [28].

Medication possession ratio (MPR) is generally considered a measure of adherence. However, we included it as a measure of retention because MPR measures *primary* adherence (indicating that the prescription was filled), rather than *secondary* adherence (indicating that the medication was taken as directed) [29]. Primary adherence and retention are two distinct concepts; however, primary adherence can be seen as a bridge between retention (access to ART) and secondary adherence (taking ART)—it is an indirect measure that assumes prescription‐refilling patterns match visit attendance and medication‐taking behaviour [30]. Therefore, MPR was included in the analyses of retention as a continuous indicator of interaction with services [30]. A threshold of 80% represents optimal MPR, as current first‐line regimens (i.e. tenofovir‐lamivudine‐dolutegravir) have lower demands on adherence, allowing a lower acceptable MPR threshold [31].

*Treatment outcomes*: Viral suppression was defined as a VL ≤1000 copies/ml and undetectable as ≤50 copies/ml, as per the World Health Organization [32], but included the threshold value as VLs were rounded off on export from the PHDC. Missing VLs were considered “not suppressed” [33]. A window of 12 months before censorship (death or database closure) was used for suppression in the last year of observed follow‐up. In sensitivity analyses, denominators included those with a completed VL, and those with a completed VL considered retained in care, to account for individuals who may not have a VL conducted or documented, particularly during the COVID‐19 pandemic.

#### 2.3.2 Analysis

*Descriptions* of characteristics used counts and proportions, and continuous variables were described using medians and interquartile ranges.

*Longitudinal retention* explored cohort‐level retention over the whole period of available observation time for each individual (time from ART initiation until they were censored at death or database closure). The six measures were applied cumulatively (i.e. over the first 2−5 years from initiation). As individuals had varying observation time, retention was presented as a proportion of those with sufficient observation time for each time period (e.g. those with 5 years of observed follow‐up time to examine retention cumulatively over the first 5 years after initiation). Individuals were considered “continuously in care” for each definition of retention if they did not experience any period of disengagement from ART care during each assessed period of time. Logistic regression was used to evaluate the odds of viral suppression (in the last year of observed time for each individual) if retained by each measure.

*Agreement between measures* was evaluated with the Cohen's Kappa statistic (with k>0.6 considered substantial agreement), to account for the contribution of agreement by chance and provide a more robust measure of agreement [34, 35].

## 3 RESULTS

### 3.1 Summary of cohort

The cohort included 68,888 adults who sought care in Khayelitsha and/or Gugulethu (Table 1). More than two‐thirds were female, and the median age at initiation was 31 years. The median duration of observation time on ART at censoring was nearly 4 years. Those accessing care in both areas were younger, with a higher proportion of females, more pivotal events and longer follow‐up. While the majority of those who were retained and had a VL conducted were suppressed ≤1000 copies/ml in their last year of observed follow‐up (93%, 31,340/40,134), low retention rates meant this translated to only 48% of the full cohort (33,377/68,888) suppressed ≤1000 copies/ml (Supporting Information 2).

**Table 1:** Characteristics and description of the cohort of people initiating antiretroviral therapy during the universal test‐and‐treat era in Khayelitsha and Gugulethu, South Africa

		Accessed care		
Characteristic	Total cohort (*n* = 68,888)	Khayelitsha (*n* = 36,823, 53%)	Gugulethu (*n* = 28,660, 42%)	Both (*n* = 3405, 5%)
**Demographics**				
Age at ART initiation	31 [26−38]	31 [26−39]	31 [26−38]	28 [24−34]
(median [IQR] in years) Proportion under 25 years (*n* [%])	17,078 [25%]	8934 [24%]	6924 [24%]	1219 [36%]
Proportion female (*n* [%])	47,631 [69%]	25,073 [68%]	19,913 [70%]	2645 [78%]
Residence (*n* [%]):				
Khayelitsha only	28,978 [42%)	27,403 [74%]	433 [1.5%]	1142 [34%]
Gugulethu only	15,509 [23%]	555 [1.5%]	14,032 [49%]	922 [27%]
Both areas	607 [0.9%]	261 [0.7%]	128 [0.4%]	218 [6.4%]
Neither area	23,794 [35%]	8604 [23%]	14,067 [49%]	1123 [33%]
**Clinical history**				
Chronic diseases^a^ (*n* [%]):	12,726 [18%]	7095 [19%]	5038 [18%]	593 [17%]
Hypertension	9033 [13%]	5097 [14%]	3579 [12%]	358 [11%]
Diabetes	2530 [3.7%]	1442 [3.9%]	986 [2.4%]	102 [3.0%]
Mental health diagnosis	3413 [5%]	1918 [5%]	1272 [4.4%]	224 [7%]
Pivotal events^a^ (*n* [%]):	36,777 [53%]	18,426 [50%]	16,067 [56%]	2284 [67%]
Pregnancy as a proportion of females	20,072/47,631 [42%]	9621/25,073 [38%]	9015/19,913 [45%]	1436/2645 [54%]
Tuberculosis	12,579 [18%]	6236 [17%]	5674 [20%]	669 [20%]
Hospital admission	16,835 [24%]	8497 [23%]	7184 [25%]	1154 [34%]
COVID‐19 diagnosis	2785 [4.0%]	1543 [4.2%]	1051 [3.7%]	191 [5.6%]
Death (*n* [%])	1957 [2.8%%]	1044 [2.8%]	825 [2.9%]	88 [2.6%]
**HIV history**				
Death (*n* [%])	1957 [2.8%%]	1044 [2.8%]	825 [2.9%]	88 [2.6%]
HIV history				
Observation time since ART initiation (median [IQR] in years)				
Proportion with a minimum duration of observation time on ART (*n* [%])	3.99 [2.75−5.08]	3.99 [2.74−5.10]	3.93 [2.70−5.02]	4.41 [3.26−5.32]
≥1 year	67,978 [99%]	36,293 [99%]	28,299 [99%]	2286 [99%]
≥2 years	59,112 [86%]	31,499 [86%]	24,481 [85%]	3132 [92%]
≥3 years	48,482 [70%]	25,860 [70%]	19,878 [69%]	2744 [81%]
≥4 years	34,295 [50%]	18,329 [50%]	13,904 [49%]	2062 [61%]
≥5 years	18,608 [27%]	10,128 [28%]	7314 [26%]	1166 [34%]
≥6 years	1351 [2.0%]	744 [2.0%]	525 [1.8%]	82 [2.4%]
ART initiation year (median [IQR])	2018 [2017−2019]	2018 [2017−2019]	2018 [2017−2019]	2018 [2017−2019]
Regimen at ART initiation (*n* [%])				
NNRTI‐based	53,914 [78%]	29,154 [79%]	21,891 [76%]	2869 [84%]
PI‐based	790 [1.1%]	441 [1.2%]	312 [1.1%]	37 [1.1%]
INSTI‐based	12,515 [18%]	6679 [18%]	5428 [19%]	408 [12%]
Unknown	1669 [2.4%]	549 [1.5%]	1029 [3.6%]	91 [2.7%]
Regimen at censoring: death or database				
closure (*n* [%])				
NNRTI‐based	23,108 [34%]	11,688 [32%]	10,277 [36%]	1143 [34%]
PI‐based	1382 [2.0%]	724 [2.0%]	579 [2.0%]	79 [2.3%]
INSTI‐based	42,608 [62%]	23,806 [65%]	16,720 [58%]	2082 [61%]
Unknown	1790 [2.6%]	605 [1.6%]	1084 [2.8%]	101 [3.0%]
CD4 within 6 months of ART initiation (*n* [%])	45,698 [66%]	24,505 [67%]	18,953 [66%]	2240 [66%]
(median [IQR])	300 [200−500]	300 [200−500]	300 [200−500]	300 [200−500]

Abbreviations: ART, antiretroviral therapy; INSTI, integrase strand transfer inhibitor; IQR, interquartile range; *n*, number; NNRTI, non‐nucleoside reverse transcriptase inhibitor; PI, protease inhibitor.

^a^ Chronic disease diagnoses and pivotal events are relative to ART: either at ART initiation or occurring thereafter, in treatment‐experienced individuals.

### 3.2 Longitudinal retention by different measures

Retention in ART care was evaluated using each measure outlined in Figure 1 (overview in Supporting Information 3a). Similar trends were found when evaluating retention with broader HIV services, combining pharmacy‐refill visits with laboratory test dates and ART clinic visits (Supporting Information 3b).

#### 3.2.1 Lost to follow‐up

Nearly half the cohort (40%, 27,620/68,888) was considered LTFU at some point, with 15% (10,187/68,249) LTFU in the first 6 months after ART initiation (Supporting Information 3a).

#### 3.2.2 Fixed‐point retention in care

**Figure 2:**
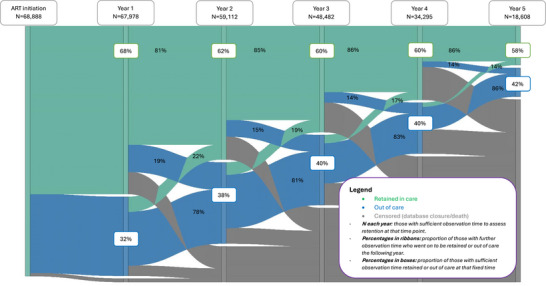
Retention in care using fixed‐point retention (with a 90‐day window either side) over the first 5 years after antiretroviral therapy initiation, for those with sufficient follow‐up at each year after initiation.

Retention in care was highest at 6 months after initiating ART (78%, 53,055/68,249 with sufficient observed follow‐up), dropping each year thereafter (Figure 2 and Supporting Information 3a). Nearly two‐thirds (62%, 42,529/68,888) were retained at censoring (death or database closure), and most (85% 1672/1957) of those who died were retained at the time of their death. Between 14% and 19% of those retained in care at each time point dropped out of care in the following year, decreasing over time from initiation. Conversely, 14−22% of those out of care returned to care in the following year (Figure 2).

#### 3.2.3 Visit constancy

The median number of visits per 6‐month window was 2.82 (interquartile range [IQR] 2.56−3.12), and median visit constancy was 75% (IQR 33–100). Both declined from the first window after initiation to the last year of observation time (Supporting Information 3a).

#### 3.2.4 Visit gaps

**Figure 3:**
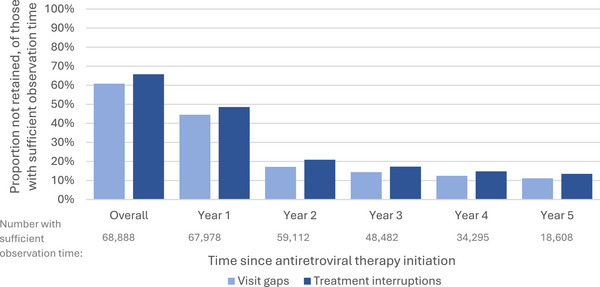
Frequency and timing of visit gaps (light blue) and treatment interruptions (dark blue) per year, considering lost to follow‐up as a gap (in those with sufficient observation time).

Nearly two‐thirds (61%, 41,908/68,888) had a gap >180 days between visits, with a median of one gap (IQR 1−1, range 1–5) in those who had a visit gap. Nearly half had a visit gap in their first year of treatment (45%, 30,281/68,888), with the proportion declining over time in those with sufficient observation time (Figure 3). More than a third (34%, 23,405/68,249) had a visit gap starting in the first 6 months after ART initiation.

#### 3.2.5 Treatment interruptions

Two‐thirds (66%, 45,310/68,888) had a treatment interruption, where they were >90 days late after an expected visit and/or LTFU over the course of their observed follow‐up, with nearly half interrupting in their first year of treatment (49%, 33,003/68,888, Figure 3). More than a third (37%, 24,960/68,249) had a treatment interruption that started in their first 6 months after initiation. The median number of treatment interruptions in those who had an interruption was 1 (IQR 1–2, range 1–11).

#### 3.2.6 Medication possession ratio

**Figure 4:**
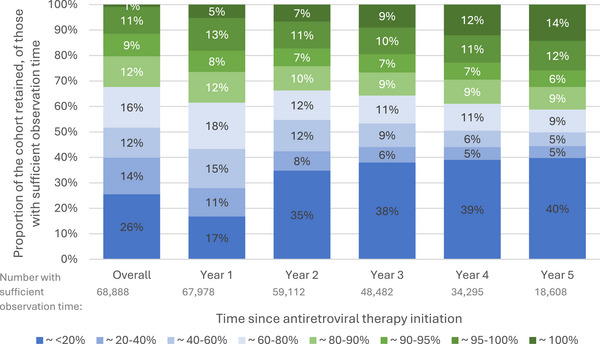
Proportion of individuals in each medication possession ratio category by year after antiretroviral initiation (in those with sufficient observation time).

The average MPR overall was low (median of 57%, IQR 19–87), and decreased from 82% (IQR 50–98) in year 1 to 51% (IQR 0–89) in year 3, and up to 60% (IQR 0–96) in year 5 after initiation (Supporting Information 3a). However, as shown in Figure 4, even with the same denominator (those with sufficient observation time in each time period), the proportion of individuals with an optimal level of adherence (≥80% ∼ green) increased in subsequent years after initiating ART. At the same time, the proportion with very low MPR (<20% ∼ blue) also increased over time, likely reflecting the accumulation of PWH who were LTFU. Supporting Information 3c describes the absolute numbers.

### 3.3 Comparison of measures of retention in ART care

#### 3.3.1 Retention continuity across measures

In general, the proportion continuously retained was low (Table 2). In those who had sufficient observation time to be evaluated, the proportion who remained continuously retained decreased with cumulative observation time.

**Table 2:** Comparison of categorization as continuously retained or not continuously retained, by different measures

		Proportion continuously retained^a^ (*n* [%])					
	Number with sufficient observation time to be evaluated *n* [%]	Not lost to follow‐up *No LTFU starting in that time period*	Fixed‐point retention in care *Retained at every time point*	100% visit constancy *All 6‐month windows have ≥1 visit*	In care by visit gaps *No gap/LTFU*	In care by treatment interruption *No interruption/LTFU*	Optimal medication possession ratio *Average MPR ≥80%*
*Overall (over the whole observation period)*	68,888	41,268 [60%]	34,510 [50%]	23,604 [34%]	26,980 [39%]	23,578 [34%]	22,381 [32%]
By cumulative years of observed follow‐up time after antiretroviral therapy initiation							
*Over 1 year*	67,978 [99%]	53,968 [79%]	46,242 [68%]	48,673 [72%]	37,697 [55%]	34,965 [51%]	26,156 [38%]
*Over 2 years*	59,112 [86%]	42,871 [73%]	32,977 [56%]	32,539 [55%]	26,565 [45%]	23,629 [40%]	18,955 [32%]
*Over 3 years*	48,482 [70%]	32,709 [67%]	24,013 [50%]	23,289 [48%]	19,222 [40%]	16,659 [34%]	14,434 [30%]
*Over 4 years*	34,295 [50%]	21,775 [63%]	15,688 [46%]	15,018 [44%]	12,480 [36%]	10,568 [31%]	9631 [28%]
*Over 5 years*	18,608 [27%]	11,074 [60%]	7925 [43%]	7521 [40%]	6278 [34%]	5235 [28%]	5117 [27%]

Abbreviations: LTFU, lost to follow up; MPR, medication possession ratio; *n*, number.

^a^ Continuously retained: Individuals were considered “continuously retained” if they were considered in care by each measure throughout their observed follow‐up.

Legend: 20–39%, 40–59%, 60–79%.

#### 3.3.2 Association of retention with treatment outcomes

Continuous retention was associated with higher odds of viral suppression ≤1000 copies/ml for all measures (Table 3). There was also a strong association with a suppressed VL in each year of observed follow‐up, and with suppression a year later (as a proxy for sustained treatment success, Supporting Information 4a).

**Table 3:** Odds of viral suppression ≤1000 copies/ml in the last year of follow‐up compared to non‐suppression, for each measure of retention

Considered in care for each measure of retention (overall over the observation period)	Odds of viral suppression ≤1000 copies/ml in the last year of each individual's observed follow‐up time OR [95% CI]
Not lost to follow‐up	56.04 [51.67−60.87]
Fixed‐point retention in care *Retained at every time point*	10.93 [10.45−11.43]
100% visit constancy *All 6‐month windows have ≥1 visit*	11.39 [10.90−11.90]
In care by visit gaps *No gap/loss to follow‐up*	10.04 [9.58−10.52]
In care by treatment interruption *No interruption/loss to follow‐up*	8.66 [8.25−9.09]
Optimal medication possession ratio *Average MPR ≥80%*	11.57 [11.06−12.11]

Abbreviations: CI, confidence interval; MPR, medication possession ratio; OR, odds ratio.

#### 3.3.3 Direct comparison between measures of retention

Of the total cohort, 23% (15,678/68,888) were considered continuously in care for their entire observed follow‐up, and 36% (24,655/68,888) were considered out of care at some point in time after initiation by all measures of retention (Table 4). There was substantial overlap between each of the measures (absolute numbers in Supporting Information 4b): most of those considered in or out of care by each measure were identified as the same status by other measures. The highest degree of agreement was between treatment interruptions and visit gaps (k = 0.88), followed by treatment interruptions and MPR (k = 0.81, Supporting Information 4b).

**Table 4:** Overlap of measures identifying people as continuously in care (green) or out of care at some point in their observed follow‐up after antiretroviral therapy initiation (red)

		Proportion continuously in care over the observation period by both measures (% of total cohort)							
		**Not lost to follow‐up** (60%)	**Retained at every fixed point** (50%)	**100% visit constancy** (34%)	**In care by visit gaps** (39%)	**In care by treatment interruptions** (34%)	**Optimal MPR ≥80%** (32%)		
Proportion out of care at some point during the observation period by both measures (% of total cohort)		23%						***Continuously in care by all measures***	**Proportion continuously in care over the observation period by both measures** (% of total cohort)
	**Lost to follow‐up** (40%)		46% κ = 0.63	34% κ = 0.52	39% κ = 0.60	34% κ = 0.52	32% κ = 0.47	**Not lost to follow‐up** (60%)	
	**Not retained by fixed‐point retention** (50%)	36% κ = 0.63		33% κ = 0.63	38% κ = 0.73	33% κ = 0.64	32% κ = 0.61	**Retained at every fixed‐point** (50%)	
	**<100% visit constancy** (66%)	40% κ = 0.52	49% κ = 0.63		31% κ = 0.75	27% κ = 0.69	26% κ = 0.66	**100% visit constancy** (34%)	
	**Visit gaps** (61%)	40% κ = 0.60	49% κ = 0.73	58% κ = 0.75		34% **κ = 0.88**	31% κ = 0.78	**In care by visit gaps** (39%)	
	**Treatment interruptions** (66%)	40% κ = 0.52	49% κ = 0.64	59% κ = 0.69	61% κ = 0.88		29% κ = 0.81	**In care by treatment interruptions** (34%)	
	**Sub‐optimal MPR <80%** (68%)	41% κ = 0.47	49% κ = 0.61	59% κ = 0.66	59% κ = 0.78	62% κ = 0.81		**Optimal MPR ≥80%** (32%)	
	***Out of care at some point by all measures***	36%							
		**Lost to follow‐up** (40%)	**Fixed‐point retention** (50%)	**<100% visit constancy** (66%)	**Visit gaps** (61%)	**Treatment interruptions** (66%)	**Sub‐optimal MPR <80%** (68%)		
		**Proportion out of care at some point during the observation period by both measures** (% of total cohort)							

Abbreviations: κ, Cohen's Kappa test statistic; MPR, medication possession ratio.

Proportion out of care at some point during the observation period by both measures

Proportion continuously in care over the observation period by both measures

## 4 DISCUSSION

### 4.1 Overview

Long‐term retention in care was suboptimal in this large cohort initiating ART in a low‐resource, high HIV‐burden setting during the universal test‐and‐treat era. The analysis of continuous retention over time demonstrated that most people were out of care at some point during their observed follow‐up. All measures were strongly associated with VL outcomes, but there was variable overlap between measures. Some retention measures, like treatment interruptions, were more stringent in determining continuity in care and provided more detailed insight into patterns of retention, while others operated more bluntly, masking the dynamic nature of retention. Measures like treatment interruptions, therefore, function as more granular tools for assessing retention over time.

### 4.2 Cohort retention in ART care: insights from measuring retention continuity using longitudinal routine data

Across all measures of retention, disengagement from ART care was common, with high proportions of people out of care at some point in time after ART initiation. These gaps matter, as continuous retention was unsurprisingly associated with viral suppression [36].

Interaction with services generally declined with longer cumulative time on treatment. A recent meta‐analysis found similar results across low‐ and middle‐income settings, with poor retention rates (defined by LTFU) that worsened over time from ART initiation. This study's rates of LTFU in Khayelitsha and Gugulethu were slightly higher than the pooled estimates (79% vs. 73% at 1 year, 73% vs. 73% at 2 years, 67% vs. 60% at 3 years and 63% vs. 49% at 4 years after initiation), possibly influenced by the PHDC data's ability to link data across facilities in the province and account for local silent transfers, a challenge noted with the data contributing to the meta‐analysis [37]. Facilities over the study period had substantial support from implementing NGOs, which may also contribute to better retention rates despite many contextual challenges to retention. However, even though this setting performed better than other low‐ and middle‐income settings, retention was still suboptimal to achieve the UNAIDS target of ending the AIDS epidemic as a threat by 2030 [38].

The MPR demonstrated a growing proportion of people doing both well (MPR >80%) and poorly (MPR <20%) over time; some people do better with longer follow‐up, and some do worse. These longitudinal dynamics are not captured by average or cross‐sectional measures. Measuring retention over time reveals a more complex pattern of interruptions and re‐engagement behind the cross‐sectional numbers [39]. More sophisticated analyses of longitudinal data could help explore these patterns and the implications for service delivery. Evaluating continuity of care, and examining it using different measures of retention, is important to complement cascade reports [40].

### 4.3 Comparison of measures of retention in ART care

#### 4.3.1 Each measure of retention compensates for limitations of other measures

No universal gold standard currently defines “adequate retention” or how to measure it [8]. While LTFU divides the cohort into those currently *in* or *out* of ART care, it only reflects one time point tethered to an arbitrary reporting date, and does not consider the pattern of retention until that point. Fixed‐point retention tracks the movement of PWH in and out of care over time. Visit constancy expands the windows around the fixed‐time points so that they continuously cover the observed follow‐up period and can identify visit regularity and patterns. However, an individual could have a visit at the start of one window and the end of the next, and be considered continuously retained, missing periods when they were out of care. Visit gaps remedy this by measuring the time between recorded visits rather than specific time points. However, individuals receive differing durations of ART refills (particularly in differentiated service delivery models with multi‐month dispensing), so that a 180‐day visit gap has different implications for someone on monthly versus 6‐monthly ART. Treatment interruptions address this using refill durations, so that an individual is only considered out of care once they have been without treatment for more than 90 days. However, this does not consider shorter, more frequent gaps in treatment that are identified when the MPR drops below 100%. These measures can, therefore, be arranged on a scale of most blunt (LTFU), through increasing granularity (fixed‐point retention, visit constancy, visit gaps and treatment interruptions) to the most granular (MPR).

#### 4.3.2 Measures have different strengths

The more granular the measure, the lower the proportion considered continuously in care. While MPR is a nuanced measure of engagement with services (retention), it is complex to calculate. Treatment interruptions had the highest agreement rating with other measures. Treatment interruptions are also an easily implementable measure by both healthcare providers (calculation from clinical notes) and researchers or programme managers (calculation from routine pharmacy‐refill data when available) [4].

Accurate identification of disengagement from ART care is important to assess facility retention success, evaluate progress towards ART coverage targets and to direct intervention [41]. However, blunter measures missed many people who were actually out of care: nearly half the individuals considered continuously retained by LTFU, 34% by fixed‐point retention, and 21% by visit constancy, were actually found to have a substantial treatment interruption of >90 days in this study. Missing these individuals means that many who need intervention would not be identified.

The choice of measure ultimately depends on the purpose of the evaluation, and the data available [8]; while treatment interruptions are an easily calculable granular measure, they require pharmacy‐refill data to calculate, compared to blunter measures like visit gaps, which only require visits and can be calculated from a broader range of data sources. Treatment interruptions must also be interpreted within their limitations: they assume that the individual would take medication until they ran out, which in reality is not always the case as people may miss doses over a longer period of time, restart the treatment they have on hand after an early interruption and return when they run out of ART, or never stop treatment at all, using stockpiled pills, borrowing medication, or attending other facilities to avoid missing doses [42, 43, 44]. We recommend presenting multiple measures of retention to improve comparability across settings with different sources of data—that is using blunter measures like visit gaps that can be compared to findings from settings without pharmacy‐refill data, as well as presenting more granular metrics (such as treatment interruptions and MPR) to make the most of data when it is available.

### 4.4 Strengths and limitations

In addition to describing retention over time from ART initiation, this study directly compared six commonly used measures, which could permit translation between programmes and studies using different measures of retention. Thresholds were aligned with local guidelines and were chosen to make the measures more comparable, but different thresholds may have performed differently. A particular strength of this analysis is the large cohort of longitudinal data from a lower‐resource setting. A recent systematic review found very few studies reporting retention past 12 months after initiation in such settings [37], making the outcomes to 5 years in this study a valuable contribution to the literature. The data from the PHDC includes interactions with all facilities in the province, so it accounts for local “silent transfers”—resulting in hopefully more accurate estimates of retention. Those out of care in this analysis were either alive but out of care, died without linkage to this data, or moved out of the province where the PHDC data cannot track them. Deaths are largely accounted for in this analysis, but many people from Khayelitsha and Gugulethu travel frequently between the Western Cape and the neighbouring Eastern Cape Province [45], and this may account for some who were considered out of care. However, a meta‐analysis of tracing studies in African ART programmes found that most noted as disengaged remained out of care [46]. In practice, many are turned away even if they do attempt to re‐engage without an official transfer letter [47], so most of those not retained in this analysis were likely truly out of care.

The COVID‐19 pandemic, which covered a large proportion of the observed follow‐up time, had substantial potential to influence retention in care as it disrupted service delivery, reduced access to care and increased the number of challenging influences on individuals’ engagement behaviour. Adaptations helped the Western Cape manage the impact of COVID‐19 on HIV services, such as mass‐switching of patients to the more robust tenofovir, lamivudine, dolutegravir combination (of which the province had more substantial stocks) and dispensing longer refills. Both the pandemic and the response may have influenced engagement with services as well as the measures of retention (e.g. for measures that could not account for longer refills).

Lastly, Khayelitsha and Gugulethu are similar settings but have substantial barriers to retention not present in all contexts (e.g. high rates of work‐related mobility) that may reduce the generalizability of these results. Additionally, the available routine health data is a unique resource with individual‐level data linking facility visit and pharmacy‐refill data across the province, which are not necessarily available for measuring retention in other settings.

## 5 CONCLUSIONS

Those continuously retained in care had high levels of treatment success, but retention was suboptimal: most PWH in Khayelitsha and Gugulethu who initiated ART in the era of universal test‐and‐treat had a gap in care over the course of their treatment. Retention is the bottleneck to increasing population‐level viral suppression and so, a target for intervention to achieve the UNAIDS 95‐95‐95 targets and control the HIV epidemic.

Measuring the continuity of retention over time helps to understand the patterns underlying poor retention and can complement cross‐sectional cascades. More sophisticated, nuanced longitudinal analyses are warranted to explore these patterns. The granular measures of retention, such as treatment interruptions, were more stringent in identifying both people in and out of care. Blunter measures like LTFU not only misassigned individuals’ retention status, but also missed the patterns of retention over time as people cycle in and out of care between points of measurement. While the recommendation is to choose as granular a measure as possible, ultimately the choice of measure depends on the purpose of the evaluation and on the data available.

## COMPETING INTERESTS

There are no relevant financial or non‐financial conflicts of interest to declare.

## AUTHOR CONTRIBUTIONS

Conceptualization: CMK, CO, JMcK and ME. Methodology: CMK, JE, TKP and CO. Data curation and formal analysis: CMK and JE. Writing—original draft: CMK. Writing—review and editing: CO, JE, TKP, JMcK and ME.

## FUNDING

This research was conducted as part of a PhD undertaken by CMK at the University of Oxford. It was supported through a scholarship from the Clarendon Fund and St John's College Kendrew Clarendon Award, in partnership with the Nuffield Department of Clinical Medicine.

## PROTOCOL, ETHICS APPROVAL AND PATIENT CONSENT

The protocol is available on the Oxford University Research Archive: https://ora.ox.ac.uk/objects/uuid:b8d73cf3‐a6e0‐4ae0‐ac54‐f36016397366

Ethical approval for this study was obtained from the University of Cape Town's Human Research Ethics Committee (066/2022). Individual patient consent was not obtained, as data was anonymized on export from the routine data centre.

## Supporting information




**Figure S1**: Flow diagram of cohort eligibility and data cleaning decisions.
**Table S1**: Datasets from the Provincial Health Data Centre that are included in these analyses.
**Table S2**: Viral suppression each year of follow‐up after initiation of antiretroviral therapy using different thresholds of suppression.
**Table S3**: Measures of population engagement, as a proportion of those with sufficient follow‐up time.
**Table S4**: Sensitivity analysis of measures of engagement using a combined dataset of pharmacy refills, laboratory visits and ART clinic visits, for those measures not solely dependent on pharmacy refill duration.
**Table S5**: Proportion with a minimum medication possession ratio at different time points.
**Table S6**: Association of measures of engagement with treatment outcomes (VL suppression ≤1000 copies/mL) in the same year.
**Table S7**: Association of measures of engagement with sustained treatment outcomes (VL suppression ≤1000 copies/mL) a year after the measure of engagement.
**Table S8**: Overlap of measures identifying people as continuously in care (green) or out of care at some point in their follow‐up (red), and the Cohen's Kappa Agreement Rating.
**Table S9**: Proportion of individuals continuously in care over the whole follow‐up period by each measure, also in care by each other measure, and the Cohen's Kappa Agreement Rating.
**Table S10**: Proportion of individuals who were considered out of care at some point during their follow‐up period by each measure, also in care by each other measure, and the Cohen's Kappa Agreement Rating.

## Data Availability

Data are available on application to the Western Cape Provincial Health Data Centre.
