# Supplementary material for: Retention in a low‐resource, high‐burden South African cohort on antiretroviral therapy: Retrospective, longitudinal analysis comparing six measures of retention
Source: J Int AIDS Soc. 2025 Oct 14;28(10):e70046. doi: 10.1002/jia2.70046 (PMC12519882; doi:10.1002/jia2.70046)
Supplement: Supplementary file 1 — Figure S1: Flow diagram of cohort eligibility and data cleaning decisions. Table S1: Datasets from the Provincial Health Data Centre that are included in these analyses. Table S2: Viral suppression each year of follow‐up after initiation of antiretroviral therapy using different thresholds of suppression. Table S3: Measures of population engagement, as a proportion of those with sufficient follow‐up time. Table S4: Sensitivity analysis of measures of engagement using a combined dataset of pharmacy refills, laboratory visits and ART clinic visits, for those measures not solely dependent on pharmacy refill duration. Table S5: Proportion with a minimum medication possession ratio at different time points. Table S6: Association of measures of engagement with treatment outcomes (VL suppression ≤1000 copies/mL) in the same year. Table S7: Association of measures of engagement with sustained treatment outcomes (VL suppression ≤1000 copies/mL) a year after the measure of engagement. Table S8: Overlap of measures identifying people as continuously in care (green) or out of care at some point in their follow‐up (red), and the Cohen's Kappa Agreement Rating. Table S9: Proportion of individuals continuously in care over the whole follow‐up period by each measure, also in care by each other measure, and the Cohen's Kappa Agreement Rating. Table S10: Proportion of individuals who were considered out of care at some point during their follow‐up period by each measure, also in care by each other measure, and the Cohen's Kappa Agreement Rating. [file JIA2-28-e70046-s001.docx]

# Supporting information

Retention in a low-resource, high-burden South African cohort on antiretroviral therapy: retrospective, longitudinal analysis comparing six measures of retention

Keene et al.

[Supporting information 1. Methodology 2](#_Toc205922848)

[Supporting information 1a. Provincial Health Data Centre datasets 2](#_Toc205922849)

[Supporting information 1b. Cohort eligibility 3](#_Toc205922850)

[Supporting information 2. Cohort treatment outcomes 4](#_Toc205922851)

[Supporting information 3 Measures of retention 6](#_Toc205922852)

[Supporting information 3a. Cohort engagement with ART using different measures 6](#_Toc205922853)

[Supporting information 3b. Sensitivity analysis of measures of engagement using all visits 7](#_Toc205922854)

[Supporting information 3c. Medication possession ratio 8](#_Toc205922855)

[Supporting information 4. Comparison of measures of retention 9](#_Toc205922856)

[Supporting information 4a. Association between retention and treatment outcomes 9](#_Toc205922857)

[Supporting information 4b. Overlap of measures of retention 11](#_Toc205922858)

## Supporting information 1. Methodology

### Supporting information 1a. Provincial Health Data Centre datasets

Table 1. Datasets from the Provincial Health Data Centre that are included in these analyses.

| Dataset | Description |
| --- | --- |
| Demographics | Wide form dataset describing demographic characteristics of the included individuals: unique study patient identifier, age at initiation, sex, residence (Khayelitsha, Gugulethu or other), drainage area (accessing care in Khayelitsha, Gugulethu or both), death status and date of death, as well as the treatment initiation date |
| Pharmacy refills | Long form dataset including each medication dispensing event, with date of refill, drug dispensed, days of drug dispensed and indication (e.g. ‘HIV’). |
| Laboratory results | All CD4 count, viral load and HbA1c laboratory results, with the date of laboratory test |
| Episodes | Long form dataset providing information on ‘episodes of care’ including hypertension, diabetes, mental health, tuberculosis, pregnancy and episodes of COVID-19. This information was combined with tuberculosis data, the maternal cascade and COVID-19 admissions data for more detail on these episodes. |
| Encounters | Long form dataset describing all encounters with the health system, including admissions, ART clinic visits, laboratory tests, pharmacy refills (HIV and other), outpatient visits, community visits and headcounts. |

### Supporting information 1b. Cohort eligibility

Data received: *all those who accessed care in Khayelitsha, Gugulethu or both and initiated antiretroviral therapy (ART) after 1^st^ January 2013*

**N=149304**

Final cohort

**N=68888**

Removed:

- <15 years old at ART initiation (n=3235)
- >85 years old at ART initiation (n=34)
- Death date < ART initiation (n=369)
- Initiated ART <1^st^ September 2016 (n=64718)
- Initiated ART > 30^th^ September 2021 (n=10601)
- Those without pharmacy refill data (n=605)
- Those with data discrepancies in ART initiation date (n=854)

Figure 1. Flow diagram of cohort eligibility and data cleaning decisions

## Supporting information 2. Cohort treatment outcomes

Table 2. Viral suppression each year of follow-up after initiation of antiretroviral therapy using different thresholds of suppression

| Threshold for suppression | Number with sufficient follow-up time as a proportion of the cohort* | Virologic suppression as a proportion of those with sufficient follow -up  (n [%]) | Viral load completed as a proportion of those with sufficient follow-up time  (n [%]) | Virologic suppression as a proportion of virologic completion (n [%]) | Retained in care as a proportion of those with sufficient follow-up time‡  (n [%]) | Viral load completed as a proportion of those retained in care (n [%]) | Virologic suppression as a proportion of virologic completion in those retained in care  (n [%]) |
| --- | --- | --- | --- | --- | --- | --- | --- |
| 1 year after initiation* | | | | | | | |
| ≤50 copies/mL | 67978 [99%] | 31032 [46%] | 37564 [55%] | 31032 [83%] | 46242 [68%] | 35872 [78%] | 30098 [84%] |
| ≤200 copies/mL |  | 33588 [49%] |  | 33588 [89%] |  |  | 32515 [91%] |
| ≤400 copies/mL |  | 34089 [50%] |  | 34089 [91%] |  |  | 32971 [91%] |
| ≤1000 copies/mL |  | 34545 [51%] |  | 34545 [93%] |  |  | 33377 [93%] |
| 2 years after initiation* | | | | | | | |
| ≤50 copies/mL | 59112 [86%] | 24630 [42%] | 29770 [50%] | 24630 [83%] | 36742 [62%] | 28589 [78%] | 24036 [84%] |
| ≤200 copies/mL |  | 26485 [45%] |  | 26485 [89%] |  |  | 25793 [90%] |
| ≤400 copies/mL |  | 26917 [46%] |  | 26917 [90%] |  |  | 26191 [91%] |
| ≤1000 copies/mL |  | 27372 [46%] |  | 27372 [92%] |  |  | 26603 [93%] |
| 3 years after initiation* | | | | | | | |
| ≤50 copies/mL | 48482 [70%] | 19706 [41%] | 23535 [49%] | 19706 [84%] | 29187 [60%] | 22636 [78%] | 19264 [85%] |
| ≤200 copies/mL |  | 20976 [43%] |  | 20976 [89%] |  |  | 20475 [90%] |
| ≤400 copies/mL |  | 21313 [44%] |  | 21313 [91%] |  |  | 20789 [91%] |
| ≤1000 copies/mL |  | 21636 [45%] |  | 21636 [92%] |  |  | 21074 [93%] |
| 4 years after initiation* | | | | | | | |
| ≤50 copies/mL | 34295 [50%] | 14388 [42%] | 16873 [49%] | 14388 [85%] | 20727 [60%] | 16323 [79%] | 14126 [87%] |
| ≤200 copies/mL |  | 15115 [44%] |  | 15115 [90%] |  |  | 14814 [91%] |
| ≤400 copies/mL |  | 15327 [45%] |  | 15327 [91%] |  |  | 15013 [91%] |
| ≤1000 copies/mL |  | 15567 [45%] |  | 15567 [92%] |  |  | 15232 [93%] |
| 5 years after initiation* | | | | | | | |
| ≤50 copies/mL | 18608 [27%] | 7417 [40%] | 8685 [47%] | 7417 [85%] | 10706 [58%] | 8419 [79%] | 7278 [86%] |
| ≤200 copies/mL |  | 7794 [42%] |  | 7794 [90%] |  |  | 7641 [91%] |
| ≤400 copies/mL |  | 7895 [42%] |  | 7895 [91%] |  |  | 7739 [92%] |
| ≤1000 copies/mL |  | 8007 [43%] |  | 8007 [92%] |  |  | 7840 [93%] |
| 6 years after initiation* | | | | | | | |
| ≤50 copies/mL | 1351 [2%] | 361 [27%] | 419 [31%] | 361 [86%] | 726 [54%] | 419 [58%] | 361 [86%] |
| ≤200 copies/mL |  | 381 [28%] |  | 381 [91%] |  |  | 381 [91%] |
| ≤400 copies/mL |  | 384 [28%] |  | 384 [92%] |  |  | 384 [91%] |
| ≤1000 copies/mL |  | 390 [29%] |  | 390 [93%] |  |  | 390 [93%] |
| Last year of observed follow-up (range 1-6 years)*†* | | | | | | | |
| ≤50 copies/mL | 68888 [100%] | 30834 [45%] | 36183 [53%] | 30834 [85%] | 40134 [58%] | 33586 [84%] | 29099 [87%] |
| ≤200 copies/mL |  | 32496 [47%] |  | 32496 [90%] |  |  | 30591 [91%] |
| ≤400 copies/mL |  | 32942 [48%] |  | 32942 [91%] |  |  | 30982 [92%] |
| ≤1000 copies/mL |  | 33377 [48%] |  | 33377 [92%] |  |  | 31340 [93%] |
| ** VL evaluated in a 12-month window (6 months either side of one year after initiation)*  *† VL evaluated in a 12-month window before database closure (30^th^ September 2022). This is <12 months in those who died within a year of initiating ART.*  *‡ Retention calculated using fixed point retention at each time point, with a window of 180 days (90 days either side of the fixed point)* | | | | | | | |

## Supporting information 3 Measures of retention

### Supporting information 3a. Cohort engagement with ART using different measures

Table 3. Measures of population engagement, as a proportion of those with sufficient follow-up time

| Time point after ART initiation | Number with sufficient follow up | Measure of engagement | | | | | | | |
| --- | --- | --- | --- | --- | --- | --- | --- | --- | --- |
|  |  | Retention | | | | | | | Adherence |
|  |  | Loss to follow-up (LTFU) | Fixed-point retention in care | | Visit attendance *Average number visits per 6 month window* | Visit constancy  *Proportion of 6 month windows that have ≥1 visit* | Visit gaps *Proportion with >180 days between visits** | Treatment interruptions  *Proportion with >90 days late for an expected visit** | Medication possession ratio  *Proportion of follow-up time with medication on hand* |
|  | N [%] | N [%] | N [%] | | Median [IQR] | Median [IQR] | N [%] | N [%] | Median [IQR] |
| *Overall*  *(over the whole follow-up period)* | **68888** | **27620 [40%]** | **42529 [62%]*** | | **2.82 [2.56-3.12]** | **75% [33-100]** | **41908 [61%]** | **45310 [66%]** | **57% [19-87]** |
| By time period after ART initiation: | | | | | | | | | |
| *First 6 months* | 68249 [99%] | 10187 [15%] | 53055 [78%] | | 4.00 [300-6.00] | NA* | 23405 [34%] | 24960 [37%] | 82% [50-98] |
| *1^st^ year* | 67978 [99%] | 14010 [21%] | 46242 [68%] | | 3.50 [2.00-4.00] | 100% [50-100] | 30281 [45%] | 33003 [49%] | 67% [33-92] |
| *2^nd^ year* | 59112 [86%] | 4910 [8%] | 36742 [62%] | | 2.00 [0.00-3.00] | 100% [0-100] | 10139 [17%] | 12344 [21%] | 51% [0-89] |
| *3^rd^ year* | 48482 [70%] | 3634 [7%] | 29187 [60%] | | 2.00 [0.00-2.50] | 100% [0-100] | 6986 [14%] | 8376 [17%] | 52% [0-91] |
| *4^th^ year* | 34295 [50%] | 2713 [8%] | 20727 [60%] | | 2.00 [0.00-2.50] | 100% [0-100] | 4263 [12%] | 5075 [15%] | 58% [0-92] |
| *5^th^ year* | 18608 [27%] | 1357 [7%] | 10706 [58%] | | 2.00 [0.00-2.50] | 100% [0-100] | 2070 [11%] | 2513 [14%] | 60% [0-96] |
| By cumulative follow-up time after ART initiation | | | | | | | | | |
| *Over 2 years* | 59112 [86%] | 16241 [27%] | | 32977 [56%] | 2.75 [1.25-3.50] | 100% [50-100] | 32547 [55%] | 35483 [60%] | 58% [24-87] |
| *Over 3 years* | 48482 [70%] | 15773 [33%] | | 24013 [50%] | 2.50 [1.00-3.17] | 83% [33-100] | 29260 [60%] | 31823 [66%] | 55% [20-85] |
| *Over 4 years* | 34295 [50%] | 12520 [37%] | | 15688 [46%] | 2.38 [1.00-3.12] | 88% [38-100] | 21815 [64%] | 23727 [69%] | 54% [19-83] |
| *Over 5 years* | 18608 [27%] | 7534 [40%] | | 7925 [43%] | 2.30 [0.90-3.00] | 80% [30-100] | 12330 [66%] | 13373 [72%] | 54% [18-83] |
|  | |  | **At database closure with 90-day window either side* | |  | **Reflects eligibility: all PWH had a first visit so had a visit in window 1* | **Considers LTFU a gap* | **Considers LTFU an interruption* |  |

### Supporting information 3b. Sensitivity analysis of measures of engagement using all visits

Table 4. Sensitivity analysis of measures of engagement using a combined dataset of pharmacy refills, laboratory visits and ART clinic visits, for those measures not solely dependent on pharmacy refill duration.

| Time point after ART initiation | Number with sufficient follow up | Measure of engagement | | | | | | |
| --- | --- | --- | --- | --- | --- | --- | --- | --- |
|  |  | Retention | | | | | | Adherence |
|  |  | Loss to follow-up (LTFU) | Fixed-point retention in care | Visit attendance *Average number visits per 6 month window** | Visit constancy  *Proportion of 6 month windows that have ≥1 visit* | Visit gaps *Proportion with >180 days between visits** | Treatment interruptions  *Proportion with >90 days late for an expected visit** | Medication possession ratio  *Proportion of follow-up time with medication on hand* |
|  | N [%] | N [%] | N [%] | Median [IQR] | Median [IQR] | N [%] | N [%] | Median [IQR] |
| *Overall*  *(over the whole follow-up period)* | **68888** | **26710 [39%]** | **47180 [68%]*** | **3.00 [2.67-3.29]** | **78% [36-100]** | **41426 [60%]** | **NA**** | **NA*** |
| By time period after ART initiation: | | | | | | | | |
| *First 6 months* | 68249 [99%] | 9293 [14%] | 54012 [79%] | 3.50 [2.00-4.50] | NA* | 22617 [33%] | NA** | NA* |
| *1^st^ year* | 67978 [99%] | 13076 [19%] | 46998 [69%] | 3.50 [2.00-4.50] | 100% [50-100] | 29626 [44%] |  |  |
| *2^nd^ year* | 59112 [86%] | 4799 [8%] | 37377 [63%] | 2.00 [0.00-3.00] | 100% [0-100] | 10462 [18%] |  |  |
| *3^rd^ year* | 48482 [70%] | 38630 [7%] | 29715 [61%] | 2.00 [0.00-3.00] | 100% [0-100] | 7316 [15%] |  |  |
| *4^th^ year* | 34295 [50%] | 2542 [7%] | 20617 [60%] | 2.00 [0.00-3.00] | 100% [0-100] | 4503 [13%] |  |  |
| *5^th^ year* | 18608 [27%] | 1414 [8%] | 10887 [59%] | 2.00 [0.00-3.00] | 100% [0-100] | 2159 [12%] |  |  |
| By cumulative follow-up time after ART initiation | | | | | | | | |
| *Over 2 years* | 59112 [86%] | NA | NA | 2.75 [1.50-3.50] | 100% [50-100] | 32115 [54%] | NA** | NA* |
| *Over 3 years* | 48482 [70%] |  |  | 2.67 [1.17-3.33] | 93% [33-100] | 28966 [60%] |  |  |
| *Over 4 years* | 34295 [50%] |  |  | 2.50 [1.12-3.25] | 88% [38-100] | 21616 [63%] |  |  |
| *Over 5 years* | 18608 [27%] |  |  | 2.40 [1.00-3.10] | 80% [40-100] | 12222 [66%] |  |  |
|  | |  | **At database closure with 90-day window either side* |  | **Reflects eligibility: all PWH had a first visit so had a visit in window 1* | ** Considers LTFU a gap* | **Considers LTFU an interruption*  ***Relies on drugs dispensed* | **Relies on drugs dispensed* |
| *Visits include:*  *- Pharmacy refills (as per main analysis) ~ median 96% of visits (IQR 90=100). Without overlap made up 883465 rows/ 1285378 rows (68.73%)*  *- Lab visits ~ median 20% of visits (IQR 15-28), with overlap with refills in a median of 14% of visits (IQR 8-20). Added 70431 rows/ 1285378 rows (5.48%)*  *- ART clinic visits ~ median of 0% of visits (IQR0-0) with overlap in a median of 0% (0-0). Added 4587 rows/ 1285378 rows (0.36%)* | | | | | | | | |

### Supporting information 3c. Medication possession ratio

Table 5. Proportion with a minimum medication possession ratio at different time points

| Time after ART initiation | Number with sufficient follow up | Medication Possession Ratio | | | |
| --- | --- | --- | --- | --- | --- |
|  |  | ≥80% | ≥90% | ≥95% | ≥100% |
|  | N [%] | N [%] | N [%] | N [%] | N [%] |
| *Overall*  *(over the whole follow-up period)* | **68888** | **22274 [32%]** | **13995 [20%]** | **7932 [12%]** | **650 [0.9%]** |
| By time period after ART initiation: | | | | | |
| *1^st^ 6 months* | 68249 [99%] | 35392 [52%] | 25207 [37%] | 21018 [31%] | 9903 [15%] |
| *1^st^ year* | 67978 [99%] | 26156 [38%] | 17960 [26%] | 12330 [18%] | 3607 [5%] |
| *2^nd^ year* | 59112 [86%] | 19934 [34%] | 14294 [24%] | 10209 [17%] | 3966 [7%] |
| *3^rd^ year* | 48482 [70%] | 17302 [36%] | 12921 [27%] | 9446 [19%] | 4370 [9%] |
| *4^th^ year* | 34295 [50%] | 13346 [39%] | 10117 [29%] | 7829 [23%] | 4127 [12%] |
| *5^th^ year* | 18608 [27%] | 7667 [41%] | 6020 [32%] | 4815 [26%] | 2649 [14%] |
| By cumulative follow-up time after ART initiation | | | | | |
| *Over 2 years* | 59112 [86%] | 18955 [32%] | 12254 [21%] | 7306 [12%] | 858 [1% |
| *Over 3 years* | 48482 [70%] | 14434 [30%] | 9079 [19%] | 5049 [10%] | 289 [1%] |
| *Over 4 years* | 34295 [50%] | 9631 [28%] | 5901 [17%] | 3247 [9%] | 90 [0%] |
| *Over 5 years* | 18608 [27%] | 5117 [27%] | 3181 [17%] | 1601 [9%] | 22 [0%] |

Considering only those who were never LTFU, 53% (22036/41268), 34% (13989/41268), 19% (7932/41268), and 1.6% (650/41268) were optimally engaged overall for the medication possession ratio thresholds of 80%, 90%, 95%, and 100% respectively over their whole follow-up period.

## Supporting information 4. Comparison of measures of retention

### Supporting information 4a. Association between retention and treatment outcomes

Association with treatment outcomes in the same year

Table 6. Association of measures of engagement with treatment outcomes (VL suppression ≤1000 copies/mL) in the same year

| Number with Sufficient follow-up time for VL outcomes (N[%]) | | Association with viral suppression ≤1000 copies/mL* (OR [95% CI]) | | | | | |
| --- | --- | --- | --- | --- | --- | --- | --- |
|  |  | Not lost to follow-up  *No LTFU starting in that time period* | Fixed point retention in care  *Retained at every time point with fixed-point retention* | 100% visit constancy  *Proportion of 6 month windows that have ≥1 visit* | In care by visit gaps  *No gap/LTFU starting in that time period* | In care by treatment interruptions  *No interruption/LTFU starting in that time period* | Optimal medication possession ratio ≥80%  *Average MPR over the time period* |
| *Overall*  *(over the whole follow-up period)* | **68888** | **56.04**  **[51.67-60.87]** | **10.93**  **[10.45-11.43]** | **11.39**  **[10.90-11.90]** | **10.04**  **[9.58-10.52]** | **8.66**  **[8.25-9.09]** | **11.57**  **[11.06-12.11]** |
| By time period after ART initiation: | | | | | | | |
| *1^st^ year* | 67978 [99%] | 12.93  [11.74-14.27] | 32.07  [9.69-34.68] | 17.18  [16.21-18.23] | 12.01  [11.46-12.59] | 8.74  [8.36-9.13] | 4.32  [4.17-4.48] |
| *2^nd^ year* | 59112 [86%] | 31.20  [27.79-35.17] | 50.53  [46.16-55.44] | 11.56  [11.05-12.10] | 10.84  [10.34-11.37] | 9.04  [8.63-9.47] | 5.29  [5.09-5.51] |
| *3^rd^ year* | 48482 [70%] | 44.83  [39.48-41.15] | 60.91  [10.93-12.66] | 13.30  [12.64-13.99] | 11.67  [11.07-12.30] | 10.05  [9.55-10.58] | 6.34  [6.08-6.63] |
| *4^th^ year* | 34295 [50%] | 55.39  [48.24-63.95] | 76.92  [67.51-88.05] | 16.15  [15.21-17.16] | 14.38  [13.49-15.34] | 12.68  [11.91-13.51] | 8.40  [7.97-8.85] |
| *5^th^ year* | 18608 [27%] | 64.00  [53.13-77.87] | 72.83  [60.63-88.35] | 15.65  [14.42-17.00] | 12.42  [11.40-13.54] | 11.11  [10.22-12.09] | 8.73  [8.14-9.36] |
| * *VL suppression in the 12 months before censorship (death or database closure), i.e. in the individual’s last year of follow-up* | | | | | | | |

Association of measures of engagement with sustained undetectable viral loads

Table 7. Association of measures of engagement with sustained treatment outcomes (VL suppression ≤1000 copies/mL) a year after the measure of engagement.

| Number with Sufficient follow-up time for VL outcomes (N[%]) | | Association with viral suppression ≤1000 copies/mL* (OR [95% CI]) | | | | | |
| --- | --- | --- | --- | --- | --- | --- | --- |
|  |  | Not lost to follow-up | Fixed point retention in care | 100% visit constancy | In care by visit gaps | In care by treatment interruptions | Optimal medication possession ratio ≥80% |
| *Overall*  *(over the whole follow-up period)* | **68888** | **56.04**  **[51.67-60.87]** | **10.93**  **[10.45-11.43]** | **11.39**  **[10.90-11.90]** | **10.04**  **[9.58-10.52]** | **8.66**  **[8.25-9.09]** | **11.57**  **[11.06-12.11]** |
| By time period after ART initiation: | | | | | | | |
| *1^st^ year* | 59112 [86%] | 461.08  [115.19-234.65] | 6.35  [6.00-6.71] | 4.05  [3.86-4.25] | 5.67  [5.42-5.94] | 4.84  [4.63-5.06] | 2.92  [2.81-3.03] |
| *2^nd^ year* | 48482 [70%] | 186.97  [137.33-263.39] | 8.8  [9.37-9.45] | 5.75  [5.49-6.02] | 6.37  [6.07-6.69] | 5.59  [5.33-5.86] | 3.74  [3.58-3.90] |
| *3^rd^ year* | 34295 [50%] | 337.46  [229.53-524.34] | 11.76  [10.93-12.66] | 7.82  [7.40-8.26] | 7.97  [7.51-8.45] | 7.07  [6.67-7.50] | 4.60  [4.38-4.84] |
| *4^th^ year* | 18608 [27%] | 377.76  [228.63-688.53] | 11.20  [10.13-12.40] | 8.15  [7.57-8.79] | 7.63  [7.04-8.27] | 6.87  [6.35-7.44] | 5.29  [4.95-5.66] |
| *5^th^ year* | 1351  [2%] | NA | 1.31  [0.77-2.23] | 7.68  [5.59-10.75] | 1.49  [1.04-2.15] | 1.27  0.90-1.81] | 4.38  [3.39-568] |
| * *VL suppression in the 12 months before censorship (death or database closure), i.e. in the individual’s last year of follow-up* | | | | | | | |

### Supporting information 4b. Overlap of measures of retention

Table 8. Overlap of measures identifying people as continuously in care (green) or out of care at some point in their follow-up (red), and the Cohen’s Kappa Agreement Rating.

|  | | Proportion continuously engaged  (N [% of total cohort] then Cohen’s κ of agreement) | | | | | |  | |
| --- | --- | --- | --- | --- | --- | --- | --- | --- | --- |
|  |  | **Not lost to follow-up** (N=41368 [60%]) | **Retained at every fixed point** (N= 34510 [50%]) | **100% visit constancy** (N= 23604 [34%]) | **In care by visit gaps** (N=26980 [39%]) | **In care by treatment interruptions**  (N=23588 [34%]) | **Optimal MPR ≥80%** (N=22381 [32%]) |  |  |
| Proportion out of care at some point during follow-up  (N [% of total cohort] then Cohen’s κ of agreement) |  | **15678 [23%]** | | | | | | ***In care by all measures*** | **Proportion continuously engaged**  (N [% of total cohort] then Cohen’s κ of agreement) |
|  | **Lost to follow-up** (N=27620 [40%]) |  | 31582 [46%]  κ = 0.63 | 23604 [34%]  κ = 0.52 | 26980 [39%]  κ = 0.60 | 23578  [34%]  κ = 0.52 | 22144 [32%]  κ = 0.47 | **Not lost to follow-up** (N=41368 [60%]) |  |
|  | **Fixed point retention** (N=34378 [50%]) | 24692  [36%]  κ = 0.63 |  | 22644 [33%]  κ = 0.63 | 26132 [38%]  κ = 0.73 | 22758  [33%]  κ = 0.64 | 21727 [32%]  κ = 0.61 | **Retained at every fixed point** (N= 34510 [50%]) |  |
|  | **<100% visit constancy** (N=45284 [66%]) | 27620  [40%]  κ = 0.52 | 33418  [49%]  κ = 0.63 |  | 21354 [31%]  κ = 0.75 | 18761  [27%]  κ = 0.69 | 17716 [26%]  κ = 0.66 | **100% visit constancy** (N= 23604 [34%]) |  |
|  | **Visit gaps** (N=41908 [61%]) | 27620  [40%]  κ = 0.60 | 33530  [49%]  κ = 0.73 | 39658  [58%]  κ = 0.75 |  | 23417  [34%]  **κ = 0.88** | 21195 [31%]  κ = 0.78 | In care by visit gaps (N=26980 [39%]) |  |
|  | **Treatment interruptions** (N=45310 [66%]) | 27620  [40%]  κ = 0.52 | 33558  [49%]  κ = 0.64 | 40467  [59%]  κ = 0.69 | 41747  [61%]  **κ = 0.88** |  | 20039 [29%]  **κ = 0.81** | **In care by treatment interruptions** (N=23588 [34%]) |  |
|  | **Sub-optimal MPR <80%** (N=46507 [68%]) | 27383  [41%]  κ = 0.47 | 33724  [49%]  κ = 0.61 | 40619  [59%]  κ = 0.66 | 40722  [59%]  κ = 0.78 | 42968  [62%]  **κ = 0.81** |  | **Optimal MPR ≥80%** (N=22381 [32%]) |  |
|  | ***Out of care by all measures*** | **24655 [36%]** | | | | | |  |  |
|  | | **Lost to follow-up** (N=27620 [40%]) | **Fixed point retention** (N=34378 [50%]) | **<100% visit constancy** (N=45284 [66%]) | **Visit gaps** (N=41908 [61%]) | **Treatment interruptions** (N=45310 [66%]) | **Sub-optimal MPR <80%** (N=46507 [68%]) |  | |
|  |  | **Proportion out of care at some point during follow-up**  (N [% of total cohort] then Cohen’s κ of agreement) | | | | | |  |  |
| *κ : Cohen’s Kappa test statistic \| MPR: Medication Possession Ratio* | | | | | | | | | |

Table 9. Proportion of individuals continuously in care over the whole follow-up period by each measure, also in care by each other measure, and the Cohen’s Kappa Agreement Rating.

|  | Proportion in care by the measure in the left column (N) | Proportion continuously engaged by the measure in the left column, also in care by the following measures (N [%] then Cohen’s κ) | | | | | |
| --- | --- | --- | --- | --- | --- | --- | --- |
|  |  | Not lost to follow-up | Fixed point retention in care | 100% visit constancy | In care by visit gaps | In care by treatment interruptions | Optimal medication possession ratio ≥80% |
| Not lost to follow-up | **41268** | **41268 [100%]** | 31582 [77%]  κ = 0.63 | 23604 [57%]  κ = 0.52 | 26980 [65%]  κ = 0.60 | 23578  [57%]  κ = 0.52 | 22144  [54%]  κ = 0.47 |
| Fixed point retention in care | **34510** | 31582 [92%]  κ = 0.63 | **34510 [100%]** | 22644 [66%]  κ = 0.63 | 26132 [76%]  κ = 0.73 | 22758  [66%]  κ = 0.64 | 21727  [63%]  κ = 0.61 |
| 100% visit constancy | **23604** | 23604 [100%]  κ = 0.52 | 22644 [96%]  κ = 0.63 | **23604 [100%]** | 21354 [90%]  κ = 0.75 | 18761  [79%]  κ = 0.69 | 17716  [75%]  κ = 0.66 |
| In care by visit gaps | **26980** | 26980 [100%]  κ = 0.60 | 26132 [97%]  κ = 0.73 | 21354 [79%]  κ = 0.75 | **26980 [100%]** | 23417  [87%]  **κ = 0.88** | 21195  [79%]  κ = 0.78 |
| In care by treatment interruptions | **23578** | 23578 [100%]  κ = 0.52 | 22758 [97%]  κ = 0.64 | 18761 [80%]  κ = 0.69 | 23417 [99%]  **κ = 0.88** | **23578 [100%]** | 20039  [85%]  **κ = 0.81** |
| Optimal medication possession ratio ≥80% | **22381** | 22144 [99%]  κ = 0.47 | 21727 [97%]  κ = 0.61 | 17716 [79%]  κ = 0.66 | 21195 [95%]  κ = 0.78 | 20039  [90%]  **κ = 0.81** | **22381 [100%]** |

Legend:

| <20% | 20-39% | 40-59% | 60-79% | >80% | Same measure |
| --- | --- | --- | --- | --- | --- |

Table 10. Proportion of individuals who were considered out of care at some point during their follow-up period by each measure, also in care by each other measure, and the Cohen’s Kappa Agreement Rating.

| Proportion out of  care by the  measure in  the left  column (N) | | Proportion out of care by the measure in the left column, also out of care by the following measures (N [%] then Cohen’s κ) | | | | | |
| --- | --- | --- | --- | --- | --- | --- | --- |
|  |  | Lost to follow-up | Fixed point retention in care | 100% visit constancy | Visit gaps | Treatment interruptions | Sub-optimal medication possession ratio ≥80% |
| Lost to follow-up | **27620** | **27620**  **[100%]** | 24692  [89%]  κ = 0.63 | 27620  [100%]  κ = 0.52 | 27620  [100%]  κ = 0.60 | 27620  [100%]  κ = 0.52 | 27383  [99%]  κ = 0.47 |
| Fixed point retention in care | **34378** | 24692  [72%]  κ = 0.63 | **34378**  **[100%]** | 33418  [97%]  κ = 0.63 | 33530  [98%]  κ = 0.73 | 33558  [98%]  κ = 0.64 | 33724  [98%]  κ = 0.61 |
| 100% visit constancy | **45284** | 27620  [61%]  κ = 0.52 | 33418  [74%]  κ = 0.63 | **45284**  **[100%]** | 39658  [88%]  κ = 0.75 | 40467  [89%]  κ = 0.69 | 40619  [90%]  κ = 0.66 |
| Visit gaps | **41908** | 27620  [66%]  κ = 0.60 | 33530  [80%]  κ = 0.73 | 39658  [95%]  κ = 0.75 | **41908 [100%]** | 41747  [99.6%]  **κ = 0.88** | 40722  [97%]  κ = 0.78 |
| Treatment interruptions | **45310** | 27620  [62%]  κ = 0.52 | 33558  [74%]  κ = 0.64 | 40467  [89%]  κ = 0.69 | 41747  [92%]  **κ = 0.88** | **45310**  **[100%]** | 42968  [95%]  **κ = 0.81** |
| Sub-optimal medication possession ratio <80% | **46507** | 27383  [59%]  κ = 0.47 | 33724  [73%]  κ = 0.61 | 40619  [87%]  κ = 0.66 | 40722  [88%]  κ = 0.78 | 42968  [92%]  **κ = 0.81** | **46507 [100%]** |

Legend:

| <20% | 20-39% | 40-59% | 60-79% | >80% | Same measure |
| --- | --- | --- | --- | --- | --- |
